# The association of human leukocyte antigen polymorphisms with disease severity and latency period in patients with silicosis

**DOI:** 10.1186/2049-6958-9-17

**Published:** 2014-03-19

**Authors:** Hasan Dogan, Metin Akgun, Omer Araz, Elif Yilmazel Ucar, Ozgur Yoruk, Eda Diyarbakir, Omer Atis, Fatih Akdemir, Hamit Acemoglu, Ibrahim Pirim

**Affiliations:** 1 Medical Biology Department, Faculty of Medicine, Ataturk University, Erzurum 25240, Turkey; 2 Chest Diseases Department, Faculty of Medicine, Ataturk University, Erzurum, Turkey; 3 Otorhinolaryngology Department, Faculty of Medicine, Ataturk University, Erzurum, Turkey; 4 Medical Education Department, Faculty of Medicine, Ataturk University, Erzurum, Turkey; 5 Medical Biology and Genetics Department, Faculty of Medicine, Katip Celebi University, Izmir, Turkey; 6 Pulmonary Diseases Department, Faculty of Medicine, Ataturk University, Erzurum 25240, Turkey

**Keywords:** HLA polymorphism, Immunity, Latency, Severity, Silicosis

## Abstract

**Background:**

Denim sandblasting may cause silicosis as a result of free crystalline silica inhalation. Its pathogenesis remains unclear, but autoimmunity may play a role in the development of silicosis. The present study aimed to investigate the relationships between human leukocyte antigen (HLA) and the severity and latency period of silicosis.

**Methods:**

48 silicotic patients in the Eastern part of Turkey were classified according to their latency period and disease severity. The distribution of HLAs according to disease severity and latency period was assessed.

**Results:**

A23 (7.5%), B49 (7.5%), and B51 (25%) were more common in the mild group than in the severe group, and B55 (8.9%) and DR4 (17.9%) were more common in the severe group than in the mild one. Only B51 was significantly more common in the mild group than in the severe one (25%, n = 10 vs. 7.1%, n = 4; p = 0.016).

**Conclusions:**

This study suggests that HLA antigens may play a particular role in the severity of silica-induced lung disease, but there was no association between HLA and progression time of the disease.

## Background

Silicosis is an ancient and well-known occupational lung disease caused by the inhalation of free crystalline silica. Certain occupations expose individuals to high concentrations of silica, which is fibrogenic to the lungs, resulting in radiological and pathological abnormalities. With time, the occupations causing silicosis have been varied. Silicosis due to denim sandblasting has recently been identified as a new cause of silicosis. Sandblasting is one of the most dangerous forms of exposure to silicosis, and it causes immediate and severe disease development [1,2]. However, there is also a variation in the latency period and severity of the disease among people working with sandblast silica-containing materials such as sifted sea sand. Many factors may play a role in the development of the disease as well as in its latency period and severity, including the respirable dust concentration in the workplace, individuals’ age, smoking habits, and use of protective equipment [3,4]. Because there is a considerable variation regarding the latency period and disease severity among the workers who operate under the same conditions, the claim that there might be an individual susceptibility to silica dust is plausible.

The Human Leukocyte Antigen (HLA) system is the Major Histocompatibility Complex (MHC) and a very polymorphic region of the genome in humans. The primary function of the HLA system is to regulate the immune response [5] and it has a big role in immunological reactions: about 100 diseases have already been associated with HLA system [6]. More specifically, the HLA complex appears to be important in the clinical expression of insulin dependent diabetes mellitus, Reiter’s syndrome, especially HLA-B27 associated ankylosing spondylitis and HLA-DR4 with rheumatoid arthritis [6,7]. It is also known that silicosis is associated with immunological reactions such as antinuclear antibody, rheumatoid factor, immune complexes, and raised immunoglobulin levels [8]. Therefore, HLA plays a role in such patients’ increasing inflammation because class I HLA molecules are located on the surface of most cells that have a nucleus and class II HLA molecules are usually found on the surface of antigen presenting cells (e.g., alveolar macrophages and B lymphocytes). Alveolar macrophages in particular play an important role in silicosis development. They are thought to be the first cells to be targeted following silica inhalation and are involved in inflammatory signals initiation [9]. HLA polymorphisms are known to play an important role in individuals’ genetic susceptibility to certain diseases. They have also been linked with immune response capability and might indicate a genetic sensitivity to silicosis in particular [8].

In this study we aimed to investigate a potential role of HLA in the development of silicosis and to address the questions of why certain individuals develop the disease and why the severity and time progression vary. In addition, our goal was to investigate the possible host factors, polymorphic regions within the HLA class I and class II typing, which contribute to the disease’s latency or severity.

## Methods

### Study population

The study included 48 male patients affected with silicosis caused by denim sandblasting, and were on follow up in the outpatient clinic of the pulmonary diseases department of our university. All the participants gave their written informed consent, and the local ethics committee of the School of Medicine, Ataturk University, approved the study protocol.

### Study procedure

The participants’ demographic data, exposure history, and clinical findings were recorded from their patient files. The latency period for each case was calculated as the time between the first exposure and the presence of the disease. Based on their radiological findings the cases were classified as having mild or severe forms of the disease according to the International Labour Office (ILO) classification system. Four main categories have been described in small opacities as (0 to 3) and 12 subcategories. A 1/0 or higher profusion was considered to be an indicator of silicosis in the radiographs. Disease severity was classified by using the ILO categories and subcategories. In further analysis a 1/1 or higher profusion was selected as a criterion of silicosis for radiographs [10,11]. The cases that had 1/1 or higher ILO profusion were classified as severe, whereas those having ILO 1/0 or lower ILO profusion without any symptoms but having proof of the disease on High Resolution Computer Tomography (HRCT) were classified as mild. All cases were also classified according to the latency period as either long (>50 months) or short (≤50 months).

### HLA DNA typing

For all patients, 2 cc of peripheral venous blood specimens were collected into test tubes including anti-coagulant (EDTA). The isolation of genomic DNA was done on an automatised DNA isolation device (MagNA Pure LC DNA Isolation Kit I, Roche) according to manufacturer’s instructions. Low-resolution typing for the HLA-A, −B, and -DR was performed by means of the PCR-sequence specific primer (PCR-SSP) method using the Micro SSP HLA class I and II generic DNA Typing Tray, Lot 008 and (One Lambda, Canoga Park, CA, USA) according to the manufacturer’s instructions. The distribution of HLA alleles according to the disease severity and latency period was compared.

### Statistical analysis

The Pearson’s Chi Square test or Fisher’s Exact test were performed to compare categorical values. All statistical calculations were performed using the SPSS 18 Program (SPSS Inc., Chicago, IL, USA) for Windows software. Statistical significance for all analyses was accepted at a level of p < 0.05.

## Results

Patients were 24 years old on the average (range: 16–44). Their characteristics are presented in Tables 1 and 2 in relation to disease severity and latency period, respectively. We compared antigen prevalence at HLA -A, −B, and -DR loci with severity and latency period of the disease. The frequencies of HLA -ABDR alleles are shown in Table 3 for the groups with severe and mild silicosis; short and long latency times are shown in Table 4.

**Table 1:** Demographic data, exposure characteristics, and lung function tests for the severe and mild groups

	Severe group**	Mild group***	**P**	**Mean**
Subjects (n)	28	20	-	-
Age (years)	24.0 ± 9.5	23.4 ± 9.2	-	NS*
BMI	22.3 ± 2.7	22.5 ± 3.0	-	NS*
Total exposure duration (months)	35.7 ± 23.5	22.1 ± 21.1	0.004	Significant
Latency period (months)	58.1 ± 25.1	42.0 ± 30.6	0.044	Significant
Smoking (pack/years)	6.7 ± 2.3	5.9 ± 4.8	-	NS*
FEV_1_ L	3.2 ± 0.8	3.8 ± 0.5	0.003	Significant
FEV_1_% pred	80.0 ± 19.9	93.5 ± 21.1	0.003	Significant
FVC L	3.8 ± 0.9	4.6 ± 0.8	0.005	Significant
FVC% pred	81.6 ± 18.3	96.0 ± 24.9	0.004	Significant
FEV_1_/FVC%	80.0 ± 9.0	84.7 ± 7.5	-	NS*

*NS, Not significant; **Severe, the cases with 1/1 or higher ILO profusion. ***Mild, the cases with1/0 or lower ILO profusion without any symptoms, but with proof of the disease on HRCT.

**Table 2:** Demographic data, exposure characteristics, and lung function tests according to latency period

	Short latency**	Long latency***	**P**	**Mean**
Subjects n	23	25	-	-
Age (years)	22.4 ± 7.2	23.4 ± 8.5	-	NS*
BMI	21.7 ± 3.1	22.6 ± 2.6	-	NS*
Total exposure duration (months)	21.4 ± 13.9	38.0 ± 27.4	0.012	Significant
Latency period (months)	37.9 ± 17.6	73.0 ± 16.6	0.001	Significant
Smoking (pack/years)	3.9 ± 4.4	8.1 ± 6.5	0.014	Significant
FEV_1_ L	3.7 ± 0.6	3.3 ± 0.9	-	NS*
FEV_1_% pred	95.0 ± 18.7	83.4 ± 24.6	-	NS*
FVC L	4.4 ± 0.8	3.9 ± 1.0	-	NS*
FVC% pred	96.6 ± 19.3	83.5 ± 25.1	0.05	Borderline
FEV_1_/FVC%	84.3 ± 7.3	83.1 ± 9.4	-	NS*

*NS, Not significant; **Short latency, cases whose latency periods are ≤ 50 months. ***Long latency, cases whose latency periods are > 50 months.

**Table 3:** Phenotype frequencies of HLA-ABDR alleles for severe and mild groups

**HLA alleles**	Severe group (n = 28 patients)**	Mild group (n = 20 patients)**	**P=**	**Mean**
**A1**	5 (8.9%)	4 (10.0%)		NS*
**A2**	16 (28.6%)	6 (15.0%)		NS*
**A3**	5 (8.9%)	2 (5.0%)		NS*
**A11**	10 (17.9%)	6 (15.0%)		NS*
**A23**	0 (0.0%)	3 (7.5%)	0.069	Borderline
**A24**	12 (21.4%)	11 (27.5%)		NS*
**A26**	3 (5.4%)	1 (2.5%)		NS*
**A29**	1 (1.8%)	2 (5.0%)		NS*
**A30**	2 (3.6%)	3 (7.5%)		NS*
**A32**	1 (1.8%)	1 (2.5%)		NS*
**A33**	0 (0.0%)	1 (2.5%)		NS*
**A68**	1 (1.8%)	0 (0.0%)		NS*
**B7**	5 (8.9%)	3 (7.5%)		NS*
**B8**	2 (3.6%)	1 (2.5%)		NS*
**B13**	2 (3.6%)	1 (2.5%)		NS*
**B18**	3 (5.4%)	0 (0.0%)		NS*
**B27**	1 (1.8%)	0 (0.0%)		NS*
**B35**	17 (30.4%)	13 (32.5)		NS*
**B37**	1 (1.8%)	1 (2.5%)		NS*
**B38**	1 (1.8%)	0 (0.0%)		NS*
**B39**	1 (1.8%)	1 (2.5%)		NS*
**B40**	1(1.8%)	3 (7.5%)		NS*
**B41**	1(1.8%)	1 (2.5%)		NS*
**B44**	4 (7.1%)	0 (0.0%)		NS*
**B49**	0 (0.0%)	3 (7.5%)	0.069	Borderline
**B50**	2 (3.6%)	0 (0.0%)		NS*
**B51**	4 (7.1%)	10 (25.0%)	0.016	Significant
**B52**	3 (5.4%)	0 (0.0%)		NS*
**B54**	1 (1.8%)	0 (0.0%)		NS*
**B55**	5 (8.9%)	0 (0.0%)	0.062	Borderline
**B57**	2 (3.6%)	2 (5.0%)		NS*
**B64**	0 (0.0%)	1 (2.5%)		NS*
**DR3**	4 (7.1%)	2 (5.0%)		NS*
**DR4**	10 (17.9%)	2 (5.0%)	0.055	Borderline
**DR7**	5 (8.9%)	3 (7.5%)		NS*
**DR10**	2 (3.6%)	2 (5.0%)		NS*
**DR11**	12 (21.4%)	11 (27.5%)		NS*
**DR13**	4 (7.1%)	7 (17.5%)		NS*
**DR14**	5 (8.9%)	6 (15.0%)		NS*
**DR15**	9 (16.1%)	5 (12.5%)		NS*
**DR16**	5 (8.9%)	2 (5.0%)		NS*

*NS, Not significant; ** Percentages were calculated according to the allele number.

**Table 4:** Phenotype frequencies of HLA-ABDR alleles according to latency period

**HLA alleles**	Short latency (n = 23 patients)**	Long latency (n = 25 patients)**	**P=**	**Mean**
**A1**	5 (11.0%)	4 (8.0%)		NS*
**A2**	11 (24.0%)	11 (22.0%)		NS*
**A3**	4 (8.7%)	3 (6.0%)		NS*
**A11**	8 (17.4%)	8 (16.0%)		NS*
**A23**	1 (2.2%)	2 (4.0%)		NS*
**A24**	8 (17.4%)	15 (30.0%)		NS*
**A26**	2 (4.4%)	2 (4.0%)		NS*
**A29**	2 (4.4%)	1 (2.0%)		NS*
**A30**	4 (8.7%)	1 (2.0%)		NS*
**A32**	0 (0.0%)	2 (4.0%)		NS*
**A33**	1 (2.2%)	0 (0.0%)		NS*
**A68**	0 (0.0%)	1 (2.0%)		NS*
**B7**	3 (6.6%)	5 (10.0%)		NS*
**B8**	1 (2.2%)	2 (4.0%)		NS*
**B13**	3 (6.6%)	0 (0.0%)		NS*
**B18**	2 (4.4%)	1 (2.0%)		NS*
**B27**	1 (2.2%)	0 (0.0%)		NS*
**B35**	15 (32.7%)	15 (30.0%)		NS*
**B37**	1 (2.2%)	1 (2.0%)		NS*
**B38**	1 (2.2%)	0 (0.0%)		NS*
**B39**	1 (2.2%)	1 (2.0%)		NS*
**B40**	1 (2.2%)	3 (6.0%)		NS*
**B41**	2 (4.4%)	0 (0.0%)		NS*
**B44**	2 (4.4%)	2 (4.0%)		NS*
**B49**	2 (4.4%)	1 (2.0%)		NS*
**B50**	1 (2.2%)	1 (2.0%)		NS*
**B51**	8 (17.4%)	6 (12.0%)		NS*
**B52**	0 (0.0%)	3 (6.0%)		NS*
**B54**	0 (0.0%)	1 (2.0%)		NS*
**B55**	2 (4.4%)	3 (6.0%)		NS*
**B57**	2 (4.4%)	2 (4.0%)		NS*
**B64**	0 (0.0%)	1 (2.0%)		NS*
**DR3**	1 (2.2%)	5 (10.0%)		NS*
**DR4**	4 (8.7%)	8 (16.0%)		NS*
**DR7**	6 (13.1%)	2 (4.0%)		NS*
**DR10**	2 (4.4%)	2 (4.0%)		NS*
**DR11**	12 (26.2%)	11 (22.0%)		NS*
**DR13**	6 (13.1%)	5 (10.0%)		NS*
**DR14**	6 (13.1%)	5 (10.0%)		NS*
**DR15**	5 (11%)	9 (18.0%)		NS*
**DR16**	2 (4.4%)	5 (10.0%)		NS*

*NS, Not significant; **Percentages were calculated according to the allele number.

In the study, 28 out of 48 patients had severe silicosis and the remaining 20 had mild silicosis. According to the prevalence of HLA -ABDR locus antigens for the severe (28) and mild silicosis (20) groups, there was a statistically significant difference between groups (p < 0.05) (Table 3). The prevalence of B51 was 10 (25.0%) in the mild group and four (7.1%) in the severe group. This difference was statistically significant (p = 0.016). The prevalence of B55 and DR4 alleles was five (8.9%) and 0 (0.0%) in the severe and mild groups, respectively, but this difference was not statistically significant (p = 0.062). Additionally, A23 and B49 antigens were not seen in the severe group, and three (7.5%) were seen in the mild group, but this difference also was not statistically significant (p = 0.069).

The prevalences of HLA -ABDR locus antigens for patients with short and long latency times are shown in Table 4. Twenty-three out of the 48 patients had a short latency time, and the remaining 25 had a long latency time. There was no significant difference between the two groups in HLA distribution according to latency time.

## Discussion

This regional study conducted in the eastern part of Turkey examined silicosis caused by denim sandblasting with the goal of reporting its associations with class I and class II human leukocyte antigens because a role of HLA has been shown in many inflammatory diseases. Our study showed that , although A23, B49, and B51 were more common in the mild group than in the severe group , and B55 and DR4 were more common in the severe group, B51 was the only statistically significant difference between the two groups. By comparing the latency period and HLA antigens there was no statistical difference between groups. The main difference between our study and previous researches consists in that we compared HLA types according to disease severity and latency. To our best knowledge, this is the first time these comparisons are done and no previous report on this issue has been published.

In literature, although there are a few reports showing that certain HLA antigens are more common incases of silicosis, none of them has found association between silicosis and any HLAs. Reported HLA type, common or uncommon, varies among the studies. Kreiss et al. [8] reported an increased frequency of HLA-B44 and HLA-A29 among 49 silicotic subjects by comparison with 1,029 white North American individuals without silicosis. In another study, they reported that Aw19 and B18 were increased [12]. In a similar study, Gualde et al. [13] reported that there was no significant association between HLAs and silicosis among the 75 patients in their 1977 study; however, they noted a decrease in the frequency of B7. In addition, they reported an increase in B8 among silicotic patients with tuberculosis, although only in a small number of patients. They only focused on comparing the HLAs of patients with silicosis and healthy controls. However, we aimed to focus on a possible association betweenHLA type and both disease severity and latency because HLA is closely associated with immunologic reactions, which may speed up the course of silicosis.

Shih et al. [7] investigated the association between asbestosis, which is another form of pneumoconiosis like silicosis, and HLA; they reported a higher prevalence of HLA-DRw53 and DQ2 in subjects with asbestos-induced pleural fibrosis. The presence of HLA-DQ2 was significantly related to the extent and type of asbestos-induced pleural fibrosis while HLA-DRw53 was not consistently related to the type or extent of pleural disease. Importantly, they observed that HLA-A29, HLA-B44, HLA-Bw4, HLA-DRw53, and HLA-DQ2 were not associated with a significantly shorter duration or latency of asbestos exposure. This study suggests that HLA may play a role in the progression of the disease and may shorten the latency period.

In our study the increased B51 in the mild silicosis group may suggest its anti-inflammatory role. This could also be linked to a lower sensitivity to silica exposure among people who carry this antigen. However, it is difficult to conclude that HLA-B51 has a negative effect on inflammation because there is an association between Behçet disease (BD) and HLA-B51. It plays a role in the pathogenesis of this auto-inflammatory disease, which includes oral and genital ulcerations, uveitis, and skin involvement as the main inflammatory alterations, and it has been strongly associated with the disease among different ethnic groups [14]. The prevalence of the HLA-B51 antigen in ethnic groups varies from 0% to more than 25%. Populations with a high HLA-B51 prevalence (> 10%) are predominantly located in the Middle East, Greece, Turkey, Iran, and Japan [15]. While B51 is identified in an average of 15–20% of the healthy population in Turkey, in BD this ratio increased to 50–60%. It has been shown that the frequency of BD increases in populations with increased HLA-B51 [14,16]. However, according to our findings, it is difficult to reach such a conclusion because B51 occurs more frequently in the mild group.

In our study, HLA-B55 and DR4 were increased in the severe group, but the increases were not statistically significant. Ayako et al. [17] showed that the frequencies of HLA-DRB1*0406 were significantly higher in patients with silicosis (16.67%) than in healthy individuals (3.03%). In another study, HLA-B55 and DR4 were significantly increased in patients with Vogt-Koyanagi-Harada syndrome (which is a systemic disorder characterized by depigmentary inflammation of melanocyte-containing tissues in Koreans) compared with a control group [18]. For this reason, they may impact the severity of the disease.

One of the reasons for the predominance of different HLAs among the studies previously described may result from HLA polymorphisms. HLA is very polymorphic and might also exist across populations of different ethnicities [16]. The fact that HLA polymorphisms exist in different groups suggests that it is important to evaluate as many different populations as possible to determine specific protective or predisposing factors for silicosis or a range of diseases associated with inflammation [19]. This is compatible with the hypothesis that environmental factors affect the regional distribution of certain HLA alleles [20]. Therefore, we can assume that the association between class I and class II HLAs and developing silicosis may differ among ethnic groups.

The main limitations of our study were the limited number of cases and the presentation of all cases from the same local region. Studies with larger sample sizes and cases from different regions will be required to show a stronger relationship. However, we had no opportunity to conduct such study because the majority of cases with silicosis caused by denim sandblasting were from the same few regions. Exposure to silica is highly associated with the working environment and there is a tendency for work in the same places among the workers from different regions. Thus, studying workers from different ethnic groups and diverse regions would be another confounding factor because of heterogeneity of exposure, that is highly associated with the disease severity and latency.

## Conclusions

We found a potential association between HLA and the development and severity of silicosis caused by denim sandblasting but no relationship between HLA and latency period. Our results suggest that the HLA-B51 phenotypes may be associated with the slow progression of silicosis and the HLA-B55 and DR4 phenotype may be associated with the development of silica-induced lung disease. However, these associations are not particularly strong, and there are no other studies supporting our findings.

## Competing interests

The authors received no financial support for the research and/or authorship of this article.

The authors declare that they have no competing interest to the publication of this article.

## Authors’ contributions

HD, Assistant Professor Doctor, design of the study. MA, Professor Doctor, editing of the manuscript. OA, Assistant Professor Doctor, statistical analysis. EYU, Assistant Professor Doctor, acquisition of data. OY, Associate Professor Doctor, data analysis. ED, Assistant Professor Doctor, data analysis. OA, Assistant Professor Doctor, acquisition of data. FA, Assistant Professor Doctor, editing of the manuscript. HA, Associate Professor Doctor, statistical analysis. IP, Professor Doctor, design of the study. All authors read and approved the final manuscript.

## References

[B1] Akgun M Gorguner M Meral M Turkyilmaz A Erdogan F Saglam L Mirici A Silicosis caused by sandblasting of jeans in Turkey: a report of two concomitant cases J Occup Health 2005 47 346 349 10.1539/joh.47.346 16096363

[B2] Akgun M Mirici A Ucar EY Kantarci M Araz O Gorguner M Silicosis in Turkish denim sandblasters Occup Med 2006 56 554 558 10.1093/occmed/kql094 17021274

[B3] Bakan ND Özkan G Çamsari G Gür A Bayram M Açikmeşe B Çetinkaya E Silicosis in denim sandblasters Chest 2011 140 5 1300 1304 10.1378/chest.10-1856 21546437

[B4] Ozden K Araz O YilmazelUcar E Alper F Akgun M Co-existence of tuberculous meningitis and pulmonary tuberculosis in a denim sandblaster Eurasian J Med 2012 44 54 57 10.5152/eajm.2012.12 PMC4261407 25610207

[B5] Bharadwaj M Illing P Theodossis A Purcell AW Rossjohn J McCluskey J Drug hypersensitivity and human leukocyte antigens of the major histocompatibility complex Annu Rev Pharmacol Toxicol 2012 10 52 401 431 22017685 10.1146/annurev-pharmtox-010611-134701

[B6] Vairo F Portela P Salim PH Jobim M Netto C Dorneles A Mittlestadt S Jobim LF Schwartz IV Human leukocyte antigens and Gaucher disease Blood Cells Mol Dis 2013 50 3 202 205 doi:10.1016/j.bcmd.2012.10.008 10.1016/j.bcmd.2012.10.008 23158683

[B7] Shih JF Hunninghake GW Goeken NE Galvin JR Merchant JA Schwartz DA The relationship between HLA-A, B, DQ, and DR antigens and asbestos-induced lung disease Chest 1993 104 1 26 31 10.1378/chest.104.1.26 8325081

[B8] Kreiss K Danilovs JA Newman LS Histocompatibility antigens in a population based silicosis series Br J Ind Med 1989 46 6 364 369 2818968 10.1136/oem.46.6.364 PMC1009787

[B9] Liu T Li L Fu C Liu H Chen D Tang F Pathological mechanisms of liver injury caused by continuous intraperitoneal injection of silica nanoparticles Biomaterials 2012 33 7 2399 2407 10.1016/j.biomaterials.2011.12.008 22182752

[B10] International Labour Office (ILO) Guidelines for the use of the Ilo International Classification of Radiographs of Pneumoconioses Revised Edition. ILO occupational safety and health series 2000 Geneva 22 (rev)

[B11] Akgun M Araz O Akkurt I Eroglu A Alper F Saglam L Mirici A Gorguner M Nemery B An epidemic of silicosis among former denim sandblasters Eur Respir J 2008 32 5 1295 1303 10.1183/09031936.00093507 18579544

[B12] Koskinen H Tiilikainen A Nordman H Increased prevalence of HLA-Awl9 and of the phenogroup Awl9, B18 in advanced silicosis Chest 1983 83 848 852 10.1378/chest.83.6.848 6851686

[B13] Gualde N De Leobardy J Serizay B Malinvaud O HL-A and silicosis Am Rev Respir Dis 1977 116 334 336 889180 10.1164/arrd.1977.116.2.334

[B14] Pirim I Atasoy M Ikbal M Erdem T Aliagaoglu C HLA class I and class II genotyping in patients with Behçet’s disease: a regional study of eastern part of Turkey Tissue Antigens 2004 64 3 293 297 10.1111/j.1399-0039.2004.00280.x 15304011

[B15] Sakly N Boumiza R Zrour-Hassen S Hamzaoui A Ben Yahia S Amara H Khairallah M Mahjoub S Bergaoui N Ghedira I HLA-B27 and HLA-B51 determination in Tunisian healthy subjects and patients with suspected ankylosing spondylitis and Behçet’s disease Ann N Y Acad Sci 2009 1173 564 569 10.1111/j.1749-6632.2009.04756.x 19758200

[B16] de Menthon M Lavalley MP Maldini C Guillevin L Mahr A HLA-B51/B5 and the risk of Behçet’s disease: a systematic review and meta-analysis of case–control genetic association studies Arthritis Rheumatol 2009 15;61 10 1287 1296 10.1002/art.24642 PMC3867978 19790126

[B17] Ueki A Isozaki Y Kusaka M Anti-caspase-8 autoantibody response in silicosis patients is associated with HLA-DRB1, DQB1 and DPB1 alleles J Occup Health 2005 47 1 61 67 10.1539/joh.47.61 15703453

[B18] Kim MH Seong MC Kwak NH Yoo JS Huh W Kim TG Han H Association of HLA with Vogt-Koyanagi-Harada syndrome in Koreans Am J Ophthalmol 2000 129 2 173 177 10.1016/S0002-9394(99)00434-1 10682969

[B19] Magira EE Papasteriades C Kanterakis S Toubis M Roussos C Monos DS HLA-A and HLA-DRB1 amino acid polymorphisms are associated with susceptibility and protection to pulmonary tuberculosis in a Greek population Hum Immunol 2012 73 6 641 646 10.1016/j.humimm.2012.03.008 22504415

[B20] Mathieu A Cauli A Fiorillo MT Sorrentino R HLA-B27 and ankylosing spondylitis geographic distribution as the result of a genetic selection induced by malaria endemic? a review supporting the hypothesis Autoimmun Rev 2008 7 5 398 403 10.1016/j.autrev.2008.03.013 18486928

